# Dynamic findings of brain magnetic resonance imaging in a haploidentical hematopoietic stem cell transplantation recipient with cytomegalovirus ventriculoencephalitis: a case report and systematic review

**DOI:** 10.3389/fimmu.2024.1450576

**Published:** 2024-09-20

**Authors:** Nannan Li, Jing Zhao, Yinghui Liu, Yuanfeng Zhang

**Affiliations:** Department of Hematology, The Affiliated Yantai Yuhuangding Hospital of Qingdao University, Yantai, Shandong, China

**Keywords:** cytomegalovirus, encephalitis, transplantation, diffusion-weighted image, case report

## Abstract

Our case demonstrated unique cytomegalovirus (CMV) encephalitis post-haploidentical donor hematopoietic stem cell transplantation (HID-HSCT), with early findings on diffusion-weighted brain magnetic resonance imaging (MRI) in the absence of any neurologic symptoms. A 54-year-old Chinese man with acute lymphoblastic leukemia (Philadelphia chromosome-negative) underwent HID-HSCT. After HSCT, the patient developed CMV viremia and severe acute graft-versus-host disease. Recurrence of CMV viremia was observed. On day 129, brain MRI was performed to determine the cause for the intermittent fever. Diffusion-weighted imaging (DWI) revealed several bright spots in the cortex of the frontal lobes and anterior angle of the left lateral ventricle. Subsequently, he developed transplant-associated thrombotic microangiopathy, posterior reversible encephalopathy syndrome, and enlargement of lesions alongside the ventricular wall on a brain MRI series. Metagenomic next-generation sequencing (NGS) of the cerebrospinal fluid (CSF) led to the final diagnosis of CMV encephalitis. Although ganciclovir combined with foscarnet was administered, the patient’s consciousness deteriorated, followed by respiratory failure. The patient died on day 198. Additionally, we performed a systematic review to comprehensively analyze this disease. Regarding treatment, immunological therapies, including virus-specific T cells from a third donor and CMV-cytotoxic T lymphocytes, may be more effective. This case report and systematic review underscores the complexities of managing CMV ventriculoencephalitis in HSCT recipients and emphasizes the importance of early diagnosis by brain MRI and CSF polymerase chain reaction or NGS and ongoing research in improving outcomes.

## Introduction

Cytomegalovirus (CMV) infection is a serious complication in hematopoietic stem cell transplantation (HSCT) recipients. CMV encephalitis is a rare but often fatal occurrence following allogeneic-HSCT (allo-HSCT). Early diagnosis and effective therapy are paramount, especially with the limited efficacy of conventional anti-CMV drugs because of drug resistance (1–7). Screening for this complication using brain MRI may be helpful. Recently, adoptive treatment using CMV-specific cytotoxic T lymphocytes (CTLs) has emerged as a promising therapeutic approach for post-HSCT CMV infection (8, 9). Here, we report the dynamic brain magnetic resonance imaging (MRI) findings in a patient who developed CMV ventriculoencephalitis from an asymptomatic onset to a fatal outcome. Additionally, we provide a systematic review of similar cases to highlight the key diagnostic and prognostic features of this rare disease.

## Case presentation

A 54-year-old Chinese man with acute lymphoblastic leukemia (Philadelphia chromosome-negative) underwent haploidentical donor HSCT (HID-HSCT) on December 1, 2019, from his 29-year-old son. The conditioning regimen included busulfan (3.2 mg/kg/day for three days), cyclophosphamide (1.8 g/m^2^/day for two days), etoposide (20 mg/kg/day for two days), and rabbit anti-thymocyte globulin (2.5 mg/kg/day for four days). Acute graft-versus-host disease (aGVHD) prophylaxis comprised cyclosporine A (CsA), short-course methotrexate (MTX), and mycophenolate mofetil (MMF).

Nine days after HSCT, the patient developed engraftment syndrome, for which methylprednisolone (MP) was initiated at 1 mg/kg/day. On day 22, he developed EBV viremia with DNA titers of 6.41×10^3^ copies/mL in nuclear cells. Low dose of rituximab (100 mg qw for two doses) was administered for preemptive therapy and tapering of steroids. After two weeks, blood test was negative for EBV. On day 26, he experienced diarrhea, and after excluding other reasons especially infective causes from the stool culture, he was clinically diagnosed with aGVHD (grade II). Despite an increase in the MP dose to 2 mg/kg/day, the patient’s symptoms persisted. On day 30, foscarnet (PFA) was initiated to address CMV reactivation with a load < 1000 copies/mL.

On day 33, diarrhea deteriorated to 1500 mL daily with bloody stools and abdominal pain, and steroid-refractory aGVHD was suspected. After that, the anti-CD25 antibody basiliximab at 20 mg was given on days 33,36,40, and 47 as along with ruxolitinib 5 mg bid and tapering of steroids. The symptoms improved after four doses, but recurred on day 53, and one dose of mesenchymal stem cells (5×10^7^) was administered. On day 70, the patient’s diarrhea was completely controlled. Minimal residual disease and CMV were negative, and his condition was deemed sufficiently stable for discharge on day 83.

**Figure 1:**
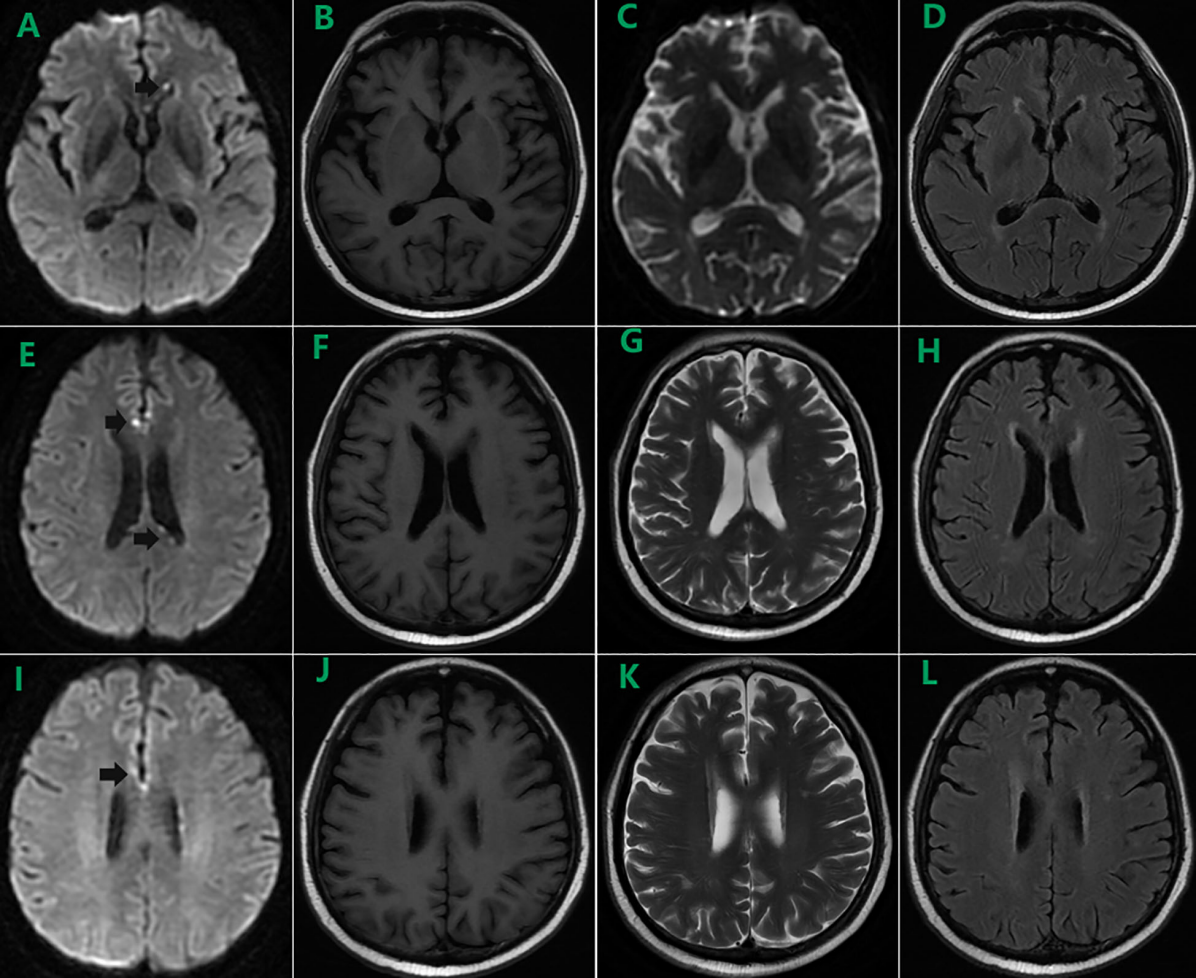
Brain MRI on day 129 showed several bright spots on diffusion-weighted image (DWI) located in the cortex of the frontal lobes and the anterior angle of the left lateral ventricle (black arrows in **A, E, I**) compared to nothing specific in the T1 **(B, F, J)**, T2 **(C, G, K)**, and T2-fluid attenuated inversion recovery (FLAIR) images **(D, H, L)**.

On day 98, the patient was readmitted owing to “diarrhea and anorexia”. Blood test revealed a recurrence of CMV infection below 1000 copies/mL with severe thrombocytopenia. After eliminating aGVHD, relapse, transplant-associated thrombotic microangiopathy (TA-TMA), and drug-induced thrombocytopenia, the low platelet count was attributed to the viral infection. Imaging was performed to identify the cause for the intermittent fever, which revealed several bright spots on diffusion-weighted image (DWI) located in the cortex of the frontal lobes and the anterior angle of the left lateral ventricle in the brain MRI (black arrows in **Figures 1A, E, I**) compared to nothing specific in the T1 (**Figures 1B, F, J**), T2 (**Figures 1C, G, K**), and T2-fluid attenuated inversion recovery (FLAIR) images (**Figures 1D, H, L**) on day 129, despite the absence of neurological symptoms (**Figure 1**). Lumbar puncture was performed with RBC and WBC counts and glucose and protein levels within normal ranges in the cerebrospinal fluid (CSF). However, tests for microorganisms, including CMV, were not performed. After treated with PFA and eltrombopag, whole blood CMV DNA became negative twice, and blood counts returned to normal levels. The patient continued to be followed in the clinic.

**Figure 2:**
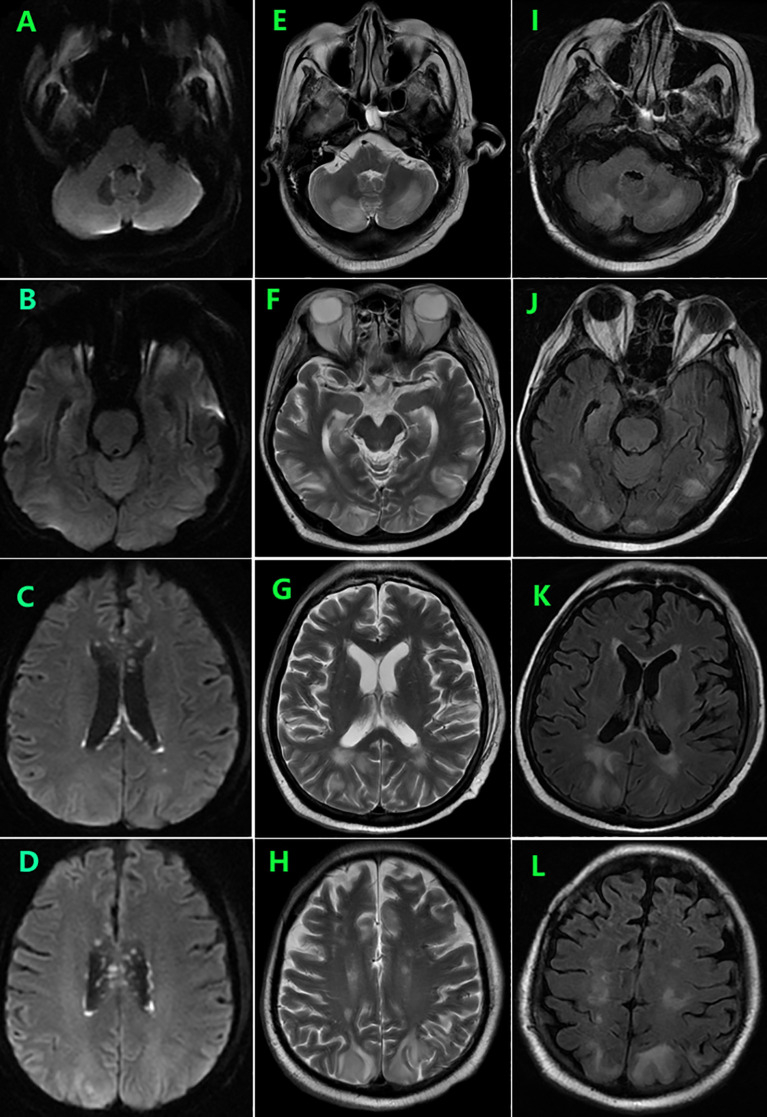
Brain MRI on day 176 showed symmetrical areas of hyperintensity in both occipital, frontal, and lobes and cerebellar involvement consistent with posterior reversible encephalopathy syndrome (PRES) **(E–L)** as well as enlargement of lesions among the lateral ventricles **(A–D)**.

**Figure 3:**
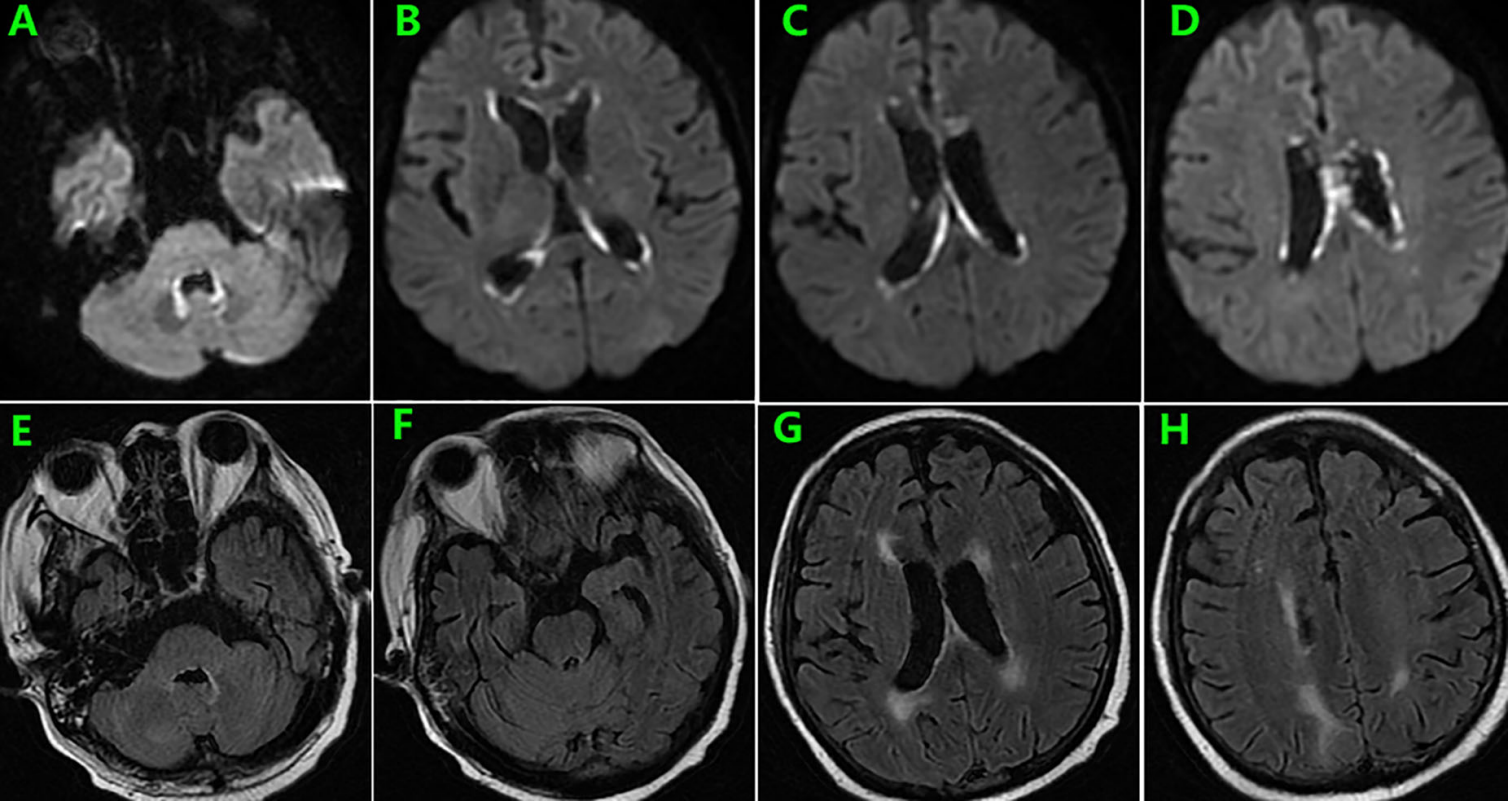
Brain MRI on day 185 showed resolution of lesions related to PRES **(E–H)** and new lesions surrounding the fourth ventricle wall as well as enlargement of former presentations along the lateral ventricles **(A–D)**.

During follow-up, the patient experienced persistent anorexia. On day 157, he reported blurred vision on the left eye, and fundus image revealed several exudates in the upper area of the retina and a hemorrhage below the macular area. Moreover, the whole blood samples were qualitatively positive for CMV DNA. On day 159, enhanced brain MRI revealed multiple abnormal punctate-signals in the anterior medulla oblongata, bifrontal cortex, and bilateral lateral ventricles. Despite ongoing antiviral therapy with foscarnet, blood CMV nucleic acid test results remained positive. On day 166, the patient experienced severely decreased hemoglobin and platelet counts, increased free hemoglobin, and repeated seizures from day 174. On day 176, brain MRI T2-FLAIR image revealed posterior reversible encephalopathy syndrome (PRES) (**Figures 2E–L**) and enlargement of lesions among the lateral ventricles (**Figures 2A–H**), suspecting TA-TMA. Treatments include plasmapheresis, discontinuation of CsA, control of CMV infection, and alternatives, including corticosteroids and MMF to prevent aGVHD. On day 185, brain MRI revealed the disappearance of lesions related to PRES and new lesions surrounding the fourth ventricle wall (**Figure 3**).

Although transient clinical improvement was achieved, the patient’s cognitive function deteriorated rapidly, manifesting as lethargy and orientation disturbance. On day 188, lumbar puncture was performed, and CSF was tested using metagenomic next-generation sequencing technology (mNGS), which detected CMV infection with 790,633 sequencing reads. CSF polymerase chain reaction (PCR) was also qualitatively positive. The patient was diagnosed with CMV ventriculoencephalitis. PFA (60 mg/kg q8h) combined with ganciclovir (GCV) (5 mg/kg q12h), and immunosuppressive drugs was discontinued. However, the patient’s condition did not improve, resulting in respiratory failure, and died on day 198. The timeline (treatment and clinical findings) of patient outcomes after transplantation is shown in **Supplementary Figure 1**.

## Systematic review

A systematic search of PubMed, Scopus, Web of Science, and Embase databases for studies published between January 1, 2020 and December 31, 2022, using the search terms “hematopoietic stem cell transplantation” or “HSCT” with “cytomegalovirus encephalitis” or “CMV encephalitis” was performed. Cases or case series meeting the following criteria were enrolled (1): patients undergoing allo-HSCT and (2) patients suffering from CMV encephalitis with details of diagnostic method, treatment, and outcomes. Patients who did not write in English were excluded.

Thirty-one studies involving 38 patients were included (**Table 1**). The patients’ median age was 29.5 years (range; 1.5–65 years). Among these patients, HID, mismatched or matched unrelated donors (MMUD or MUD), and umbilical donors (UD) accounted for 94.7% (36/38), whereas almost all patients (37, 97.4%) had previously suffered from CMV viremia. Regarding CMV serostatus, only one patient and donor were negative, whereas 24 were positive for either the patient or donor (D+/R- 3, D-/R+ 9, D+/R+ 12). Regarding anti-CMV treatment, 28 of the 38 patients received both GCV or valganciclovir (VGC) and PFA for CMV viremia. Excluding one patient with CD4+ cell >200/ul, low CD4+ cell counts during onset (range; 0–132/uL) were observed. Notably, the median number of days to CMV encephalitis onset was 180 days post-transplantation. Of the 20 patients with mutation tests in the CSF, 11 (55%) had mutations in UL97 or 54, whereas 10 of 14 patients had mutations in UL97 or 54 in the peripheral blood (PB). Notably, compartmentalization of viruses present in the PB and CSF was observed in five patients (3, 4, 10–12). Only 10 patients were alive during follow-up, with a survival rate of 26.3%; the leading cause of death was uncontrolled CMV infection. Particularly, three were recovered by GCV, PFA, and cidofovir (CDV) or VGC (13–15) while the remaining seven patients were successfully treated by virus-specific T cells (VST) from a third-party donor (n=1) (16), CMV-CTLs (n=1) (5), donor lymphocyte infusion (DLI) or immune globulin (IG) with or without drugs (n=4) (17–20), or CMV-specific IG alone (n=1) (21).

**Table 1:** Details CMV encephalitis among patients undergoing allogeneic transplantation in literature.

Ref	Age (yrs)	Sex	Diagnosis	Transplant type	CMV serostatus	Treatment of viremia	CD4^+^ count (cells/ul)	Days of encephalitis	Diagnostic method	DR in CSF	DR in PB	Treatment of encephalitis	Outcomes	COD
(1)	6	F	ALL	HID	D+/R+	GCV, PFA, and IG	10	220	CSF PCR	UL54	NA	GCV, PFA, CDV, and IG	Expired	Organ failure
(2)	20	M	MLD	MUD	D-/R+	GCV, PFA, and IG	NA	166	CSF PCR (P-M)	UL97	NA	GCV and PFA	Expired	CMV
(10)	48	F	AML	MUD	D+/R-	GCV and PFA	Low	170	CSF PCR	No	UL97	GCV and PFA	Expired	CMV
(38)	13	F	AML	MUD	NA	NA	NA	NA	NA	NA	NA	NA	Expired	Relapse
(3)	30	F	AML	HID	D+/R+	GCV and PFA	30	285	CSF PCR	UL97	UL97	GCV, PFA, and IG	Expired	CMV
(3)	54	M	AML	HID	D-/R-	GCV and PFA	NA	201	CSF PCR	No	UL97	GCV and PFA	Expired	CMV
(39)	55	M	AML	HID	D+/R+	GCV and PFA	70	137	CSF PCR	No	NA	PFA and IG	Expired	CMV
(11)	64	M	AML	MUD	NA	GCV and PFA	0	180	CSF PCR	No	UL54	GCV and PFA	Expired	CMV
(40)	46	M	DLBCL	MUD	D-/R+	GCV and PFA	NA	210	CSF PCR	NA	UL97/54	GCV, PFA, CDV, and LEF	Expired	CMV
(17)	18	F	AML	MMUD	D-/R+	Multiple drugs^*^	0	220	CSF PCR	UL97	UL97	GCV, PFA, CDV, and IG	Alive	–
(41)	53	F	AML	CB	NA	PFA	NA	148	Pathology (P-M)	NA	NA	PFA	Expired	CMV
(42)	41	M	CML	MSD	D+/R-	GCV	NA	175	CSF PCR	UL97	UL97	PFA	Lost	–
(43)	20	M	ALL	CB	NA	PFA, GCV, and IG	NA	185	CSF PCR	NA	NA	PFA and anti-CMV IG	Expired	CMV
(22)	55	M	MDS	CB	D-/R+	GCV and PFA	49	215	CSF PCR	NA	NA	GCV, PFA, and CDV	Expired	CMV
(22)	41	M	BP-AL	CB	D+/R+	GCV, PFA and VGC	43	239	CSF PCR	NA	NA	PFA, CDV, and IG	Expired	CMV
(18)	29	F	T-NHL	MMUD	D+/R+	GCV and PFA	<10	107	CSF PCR	NA	UL97	CDV, IG, LEF, and DLI	Alive	–
(19)	56	M	DLBCL	MUD	D-/R+	NA	<100	210	CSF PCR	NA	NA	GCV, PFA, and IG	Expired	CMV
(19)	44	M	HL	MUD	D+/R+	NA	<100	240	CSF PCR	NA	NA	GCV, PFA, and IG	Alive	–
(44)	20	M	ALL	CB	NA	PFA	NA	NA	MRI	NA	NA	NA	Expired	CMV
(4)	3	M	NB	HID	D+/R+	GCV and PFA	NA	181	CSF PCR	No	UL97/54	GCV, PFA and IG	Expired	CMV
(45)	4	F	ALL	HID	D+/R-	GCV and PFA	39	106	CSF PCR	No	No	GCV	Expired	Relapse
(45)	58	M	AML	MUD	D-/R+	GCV	132	396	CSF PCR	No	No	GCV, PFA and IG	Expired	CMV
(35)	28	F	MF	CB	NA	GCV and PFA	0-1	122	CSF PCR	NA	NA	GCV and PFA	Expired	CMV
(46)	65	M	DLBCL	MUD	NA	VGC and PFA	NA	NA	CSF PCR	NA	NA	Acyclovir and PFA	Expired	CMV
(46)	6	F	ALL	MSD	NA	GCV and PFA	NA	NA	CSF PCR	NA	NA	GCV, PFA, and CDV	Expired	Sepsis
(13)	11	F	SAA	MUD	D-/R+	GCV and PFA	NA	4 months	CSF PCR	UL97	NA	GCV, PFA, and CDV	Alive	–
(12)	1.8	M	IID	CB	NA	GCV, PFA and CDV	NA	27 months	CSF PCR	UL54/97	No	GCV, PFA, and VGC	Expired	Organ failure
(14)	59	M	AML	CB	NA	No	NA	38	CSF PCR	NA	NA	CDV, PFA, and GCV	Alive	–
(47)	15	M	ALL	CB	D+/R+	NA	NA	300	CSF PCR	NA	NA	GCV and PFA	Expired	CMV
(15)	59	F	FL	MUD	NA	Multiple drugs^#^	NA	113	CSF PCR	NA	No	GCV and PFA	Alive	–
(16)	1.5	F	CA	MUD	NA	GCV and PFA	NA	150	CSF PCR	NA	NA	Third party donor VST	Alive	–
(16)	8	M	FA	MUD	NA	GCV and PFA	NA	180	CSF PCR	No	NA	Third party donor VST	Expired	Adenovirus
(20)	6	M	FA	HID	D+/R+	GCV, PFA, and IG	10	105	CSF PCR	NA	NA	DLI	Alive	–
(5)	27	M	T-ALL	HID	D+/R+	NA	NA	153	CSF PCR	UL54/97	NA	Donor CMV-CTLs	Alive	–
(5)	57	F	AML	HID	D+/R+	GCV	NA	116	CSF PCR	UL54/97	NA	Donor CMV-CTLss	Expired	Septic shock
(21)	10	F	ALL	MUD	D+/R+	VGC and PFA	>200	178	CSF PCR	No	NA	CMV-IG	Alive	–
(6)	53	M	AML	HID	D-/R+	GCV, PFA, and LEF	NA	260	CSF PCR	UL54/97	UL97/54	PFA, GCV, LEF, and IG	Expired	CMV
(7)	43	M	AML	MMUD	D-/R+	VGC	NA	230	CSF PCR	UL54	NA	GCV and intrathecal IG	Expired	CMV

Ref reference, yrs years old, DR drug resistance, CSF cerebrospinal fluid, PB peripheral blood, COD cause of death, F female, ALL acute lymphoblastic leukemia, HID haploidentical donor, D donor, R receipt, GCV ganciclovir, PFA foscarnet, IG immune globulin, PCR polymerase chain reaction, NA not available, CDV cidofovir, M male, MLD, metachromatic leukodystrophy, MUD matched unrelated donor, P-M post-mortem, AML acute myeloid leukemia, DLBCL diffuse large B cell lymphma, LEF leflunomide, MMUD mismatched unrelated donor, CB cord blood, CML chronic myeloid leukemia, MDS myelodysplastic syndrome, BP-AL bi-phenotypic acute leukemia, VGC valganciclovir, DLI donor lymphocyte infusion, HL Hodgkin lymhoma, NB Neuroblastoma, MF mycosis fungoides, SAA severe aplastic anemia, FL follicular lymphoma, CA Congenital neutropenia, IID inherited immune deficiency, FA Fanconi anemia, VST virus-specific T cells, CTLs cytotoxic T lymphocytes.

* GCV, PFA, CDV, IG, and VGC; ^#^ VGC, GCV, PFA, CDV, and artesunate.

## Discussion and conclusion

CMV encephalitis is an extremely rare post-HSCT complication. In retrospective studies, the incidence ranges from 0.1%–2.3% (22, 23). However, the prognosis is poor and should not be neglected.

Several factors contribute to the development of the condition. First, impaired T cell immune function caused by intensive GVHD prophylaxis conditioning regimen including ATG or anti-CD52 antibody, severe aGVHD, and delayed immune reconstitution following UC transplantation, are crucial in its emergence. Second, CMV viremia is another risk factor. Moreover, prolonged exposure to anti-CMV drugs, such as GCV and PFA may lead to drug resistance mutations in UL97 or 54. Therefore, CMV reactivation prophylaxis with new drugs such as letermovir may reduce this fatal complication. Primary prophylaxis with letermovir has been found to be a significant beneficial factor in preventing refractory or resistant CMV infections, reducing non-relapse mortality at week 48 and clinically significant CMV infections and diseases after allo-HSCT (24). Third, compartmentalization of anti-viral sensitive CMV in the CSF may indicate an insufficient concentration of GCV, PFA, or CDV across the blood-brain barrier (11). Our case was an HID-HSCT recipient with ATG added to the conditioning regimen, severe aGVHD post-HSCT, and poor immune reconstitution marked as a low CD4+ cell count (only 37/uL). Previously, CMV reactivation was approximately 85% according to the ATG-based regimen in HID-HSCT (25–28) and CMV drug resistance was approximately 14.5% (29).

Clinically, the diagnosis of CMV encephalitis depends on CSF PCR while the typical finding of an “owl eye sign” by brain pathology is difficult to perform (30). In our case, based on a series of brain MRIs and reported literature (31), we found extremely early findings of CMV encephalitis presenting as bright spots on DWI and dynamic changes in these lesions. Our case revealed that routine MRI screens, especially DWI images, may be important in early diagnosis (31). Specifically, brain DWI and mutation detection should be performed in HSCT patients with relapsed or refractory CMV viremia in the absence of neurological symptoms. For patients with suspected encephalitis, a CSF test panel that includes CMV, human herpesvirus 6, EBV, and herpes simplex virus tests should be performed. Moreover, if CMV is a probable pathogen, CMV mutation detection both in the PB and CSF should be implemented (32–34).

Concerning treatment, despite aggressive antiviral therapy, many cases have poor prognosis (22, 35), highlighting the urgent need for novel treatment strategies. The high mortality of this disease may be ascribed to the low concentration of anti-viral drugs in the CSF, drug resistance, and poor recovery of the immune system. Patients successfully treated by anti-viral drug combinations experienced a long-term course and tapering of immunosuppressants (13–15).Successful cases of immunological therapies, including VST from a third donor (16), CMV-CTLs (5), DLI (18, 20), and CMV-specific IG (21), have been reported. In a retrospective study, six out of 10 patients with CMV encephalitis who may benefit from CMV-CTLs were salvaged (30). However, the time-consuming production of cells and the urgency of this disease may limit the application of CTLs in practice, although we may fear the potential GVHD effects of DLI. In clinical trials, the application of off-the-shelf CMV-specific CTLs has shown great benefits in treating viral reactivation (36); however, further studies are required to determine the benefits of CMV-specific T cells in the treatment of CMV-encephalitis, provided the poor understanding of the penetration of CMV-specific CTLs in the CSF.

There are several limitations to its diagnosis and treatment. First, we did not perform CSF microbiological analysis at the beginning because of the limited knowledge on this disease. Second, the compartmentalization of viruses following allo-HSCT should be focused. Third, we may consider tapering CsA rapidly to help reconstitute the immune system and administering DLI in the absence of aGVHD to treat this disease. Fourth, in our patient, as CMV was detected using a qualitative method, the quantitative level of CMV DNA in the peripheral blood was monitored regularly, not less than once weekly, and mutation detection was initiated as refractory CMV viremia was suspected after the second recurrence of CMV viremia on day 157 (37).

## Data Availability

The raw data supporting the conclusions of this article will be made available by the authors, without undue reservation.
