# Supplementary figures and images for: Dynamic findings of brain magnetic resonance imaging in a haploidentical hematopoietic stem cell transplantation recipient with cytomegalovirus ventriculoencephalitis: a case report and systematic review
Source: Front Immunol. 2024 Sep 20;15:1450576. doi: 10.3389/fimmu.2024.1450576 (PMC11449730; doi:10.3389/fimmu.2024.1450576)

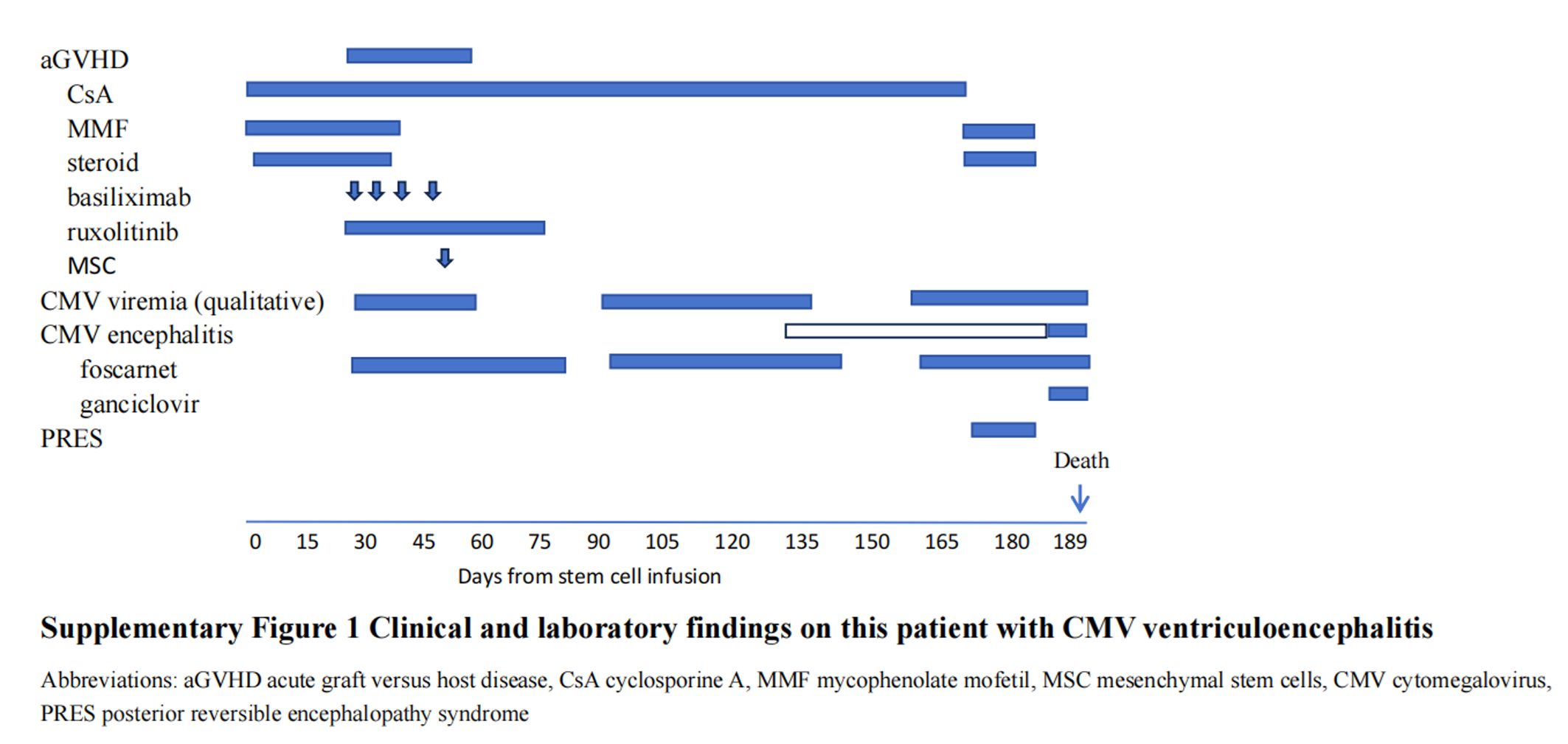

Supplement: Supplementary file 1 [file Image1.tif]
